# Mr. Grose on Yeast, in Reply to Mr. Custance

**Published:** 1801-03

**Authors:** J. H. Grose

**Affiliations:** Winslow


### *To the Editors of the Medical and Phyſical Journal.*

GENTLEMEN,

I Was much pleaſed with the peruſal of a letter inſerted in your Number for February, 1801, from the pen of Mr. Cuſtance, and intended as a reply to a former epiſtle of mine, as it affords me the gratification of again ſoliciting the public attention to a ſubject of conſiderable importance.

During

During an extenfive practice among putrid fevers, I have had many opportunities of obferving the effects of Yeaft, particularly after the medicines ufually adminiftered had loft their falutary influence; and finding among the generality of cafes, not only a more rapid but fuccefsful termination, I was pleafed with the refult, and determined, in moft inftances that occurred, to make trial of its efficacy; though, frequently, from the averfion of the patient to the naufeoufnefs of its tafte, I was neceffitated to have recourfe to the old routine, and am happy to add, not unfrequently with advantage. But in the typhus of young children, the quantity of bark that could be got down was fo fmall, as to be productive of no good effect, and among them a more powerful remedy has long been a defirable object. Whenever an opportunity occurred, I did not fail to adminifter yeaft, and can with confidence affert, among a variety of inftances, not one terminated unfavourably. Such aftonifhing fuccefs firft prompted me to infert the hiftory of a cure effected folely by this medicine; and as feveral gentlemen of undoubted veracity had already communicated a variety of cafes in which the anti-putrefcent properties of yeaft had proved beneficial, I thought it needlefs to trouble you with more, not doubting but their teftimony, in addition to mine, would excite enquiry. With thefe principles only, I ventured to intrude on your attention; I believe, I avowed my motive in a former communication; and having perufed feveral letters from gentlemen of eminence, recommending its ufe from principles of humanity, I confefs I was hurt to obferve a man of Mr. Cuftance's abilities condemn, in fo vehement a manner, what I knew from experience to be ufeful. Perhaps, a miftaken judgment might mif-conftrue his meaning; but what are we to conclude from the following lines? "Thus we fee, that however well a ftrong Dutch girl may bear *working* with yeaft, *it will not be safe* to have recourfe to fuch means in recovering our fair country-women from putrid fevers!" By *working*, I fhould fuppofe he meant to convey an idea as if jalap or gamboge had been given; and it appears plain to one poffeffed only of common fenfe, that he perfecutes this remedy, when he fays, "*it is not safe* to have recourfe to fuch means in recovering our fair country-women from putrid fevers!" I cannot help admiring the ingenuity with which he endeavours to convince you that I have mifconceived the purport of his letter, by fixing my eye too long on a particular paffage; but unlefs I am deftitute of underftanding, the words require no more than to be taken in their ufual acceptation.

Mr. C. informs us, his reafon for difapproving of yeaft was, to "obviate the danger of empiricifm." Why then did he he addrefs his letter to the Medical Journal? as I am inclined to think, this publication is but feldom perufed by perfons of that defcription, and chiefly calculated for the inftruction of men of education, who, by imparting their progrefs in the cure of difeafes, and the remedies employed, are enabled to develope many improvements, and difcard injurious preparations. Had it been inferted in the *Armenian, or Methodist's Magazine*, where, according to Mr. C. an exaggerated account firft appeared, it might, probably, from falling into the hands of the lower clafs, have been productive of fome advantage.

It is fomething fingular, that this gentleman, after expofing my inadvertency, fhould err in a fimilar manner. He proceeds: "*The statement of the case, as given by Mr. G. appears to be very vague and unsatisfactory. He tells us that the boy was attacked on the* 10*th of July with the usual symptoms of typhus. On the third day, that is, on the* 12*th, the sponging and use of yeast appear to have been begun. From the* 13*th he became evidently better: In two days after the yeast was discontinued. On consideration of the wonderful change which so rapidly succeeded the administration of the yeast, says Mr. G. I cannot but imagine it to be the best remedy in putrid fevers ever yet discovered. Are we to conclude from this relation, that Mr. G's patient was cured of a putrid fever within five days from its first attack: If I can understand Mr. G. right, this is the conclusion he wishes to be drawn.*" According to this mode of reafoning, the words "*evidently better*" are conftrued into a perfect cure; for if my letter is referred to, I made ufe of no other expreffion; I conceived the boy, at the time the yeaft was difcontinued, *free from any putrid tendency*, though the feverity of the attack made him exceedingly low for fome time after.

Mr. C. is of opinion, becaufe bark was alternately adminiftered in conjunction with yeaft, the appellation of fpecific beftowed on the latter is unjuft; the cure, fays he, may as well be attributed to the bark; and again, "*in the solitary case with which Mr. G. has favoured the public in No. XXI. the patient was sponged all over, and directed the free use of yeast and wine.*" As I am unacquainted with the circumftances of the cure recorded in the Methodift's Magazine, I cannot prefume to offer an opinion; nor do I credit the poffibility of a patient in the laft ftage of putrid fever, being feen the next morning, after taking fome yeaft, walking in his garden apparently well; but from my own knowledge I can affirm, that it has proved effectual in reftoring convalefcence, after repeated quantities of bark had failed; and furely this circumftance alone is fufficient to eftablifh its claim to fuperiority, when it raifes a fuperftructure on the failure of fo popular a medicine. Not that

I wifh

I wifh to decry the utility of bark; but it does not unfrequently happen, that a very fmall and infufficient quantity to counteract the putrid diathefis can be got down, and where it is plentifully adminiftered, even to the extent of an ounce of powder in twenty-four hours, (and no lefs can be depended upon) the tone of the ftomach is often, from a variety of caufes, and efpecially hard drinking, fo much impaired, as to be incapable of converting it to any falutary purpofe. But I am authorifed by experience to affirm, that in the far greater part of thefe inftances, where bark was wholly ufelefs, even fmall dofes of yeaft have been eminently ferviceable.

But, fays Mr. C. in the folitary cafe which I addreffed to your Journal, many of your readers will attribute the fuccefs that followed as much to the vinegar and wine as to the yeaft. This is the firft account I have ever heard of cold vinegar (or water, for their influence in affufion is precifely fimilar) actually curing putrid fever; I will quote fome part of that letter without altering the meaning, and fee how far his ideas agree with the motive that induced me to make ufe of it. After ftating the manner in which I applied it, I obferved, "*he was sponged again at night, and I saw him soon afterwards evidently better, his pulse being regular, and body cool.*" Does it not appear very confpicuous, that the intention of applying cold vinegar was to diminifh the febrile heat, and confequently promote perfpiration; and that it was ufed as an auxiliary to the remedy? On what authority this idea is grounded, I am at a lofs to imagine, for if we refer to the writings of both antient and modern practitioners, we fhall find no one inftance on record, of cold affufion curing putrid fever without the affiftance of an internal medicine; that it has removed ardent fever, and in all topical inflammations proved of fingular efficacy, I am fatisfied; but never before heard of fuch decided fuperiority in the former difeafe. If we look into the writings of the antients, we fhall find they ufed it with advantage in hot burning fevers. Hippocrates fays, if the patient is thirfty while labouring under an acute fever, cold water is of great ufe, if given till it makes him vomit. Galen fays, that cold water is a perpetual remedy, either by immerfion or drinking. Paulus obferves, that heat may be extinguifhed by cold water, by which we have wholly cured burning fevers. Celfus advifes the ufe of cold water, and directs the patient to drink to fatiety; and Paulus alfo adds, the cold bath alone is of fervice to thofe who labour under an ardent fever without an inflammation, a tumor, or an eryfipelas; and in conformity with thefe obfervations, the learned and ingenious Dr. Currie has publifhed a variety of inftances where affufion has removed the intenfe heat; but the difcovery of its folely curing putrid fever, even allowing the ufe of wine, was referved for Mr. Cuftance. Your readers will then perceive the motive of my actions; and I hope I have made it appear thus far my arguments were not wholly inconclufive. Probably, the happy termination of this folitary cafe might be owing to the wine, but this fuggeftion is equally defeafible. Let us refer to the ftatement; "from the 10*th to the* 12*th*, *that is*, *for three days*, *he took repeated quantities without the least benefit*, *and on the latter*, *the yeast and sponging were begun*; and by a proper perfeverance in their ufes for three days longer, (making in the whole a period of fix days, which Mr. C. fhortened) the boy was fo far *cured*, (if I may be allowed the expreffion) as to be out of danger. After having previoufly taken fufficient quantities of wine, I am furprized it manifefted no apparent effect, or that its anti-putrefcent properties fhould be dormant fo long, to burft forth at laft with renovated vigour. But if the generality of your readers, as Mr. C. conjectures, will attribute this providential recovery to the wine, I will humbly acquiefce, though it is plain, Mr. C. is willing to beftow the laurel on any thing but yeaft.

Mr. C. juftly obferves, I have brought forward but one inftance of its fuccefs, and this is undoubtedly a very fair objection, as it is impoffible to form any decifive opinion from the hiftory of a "*Solitary Cafe.*" As I have already ftated my motive for fo doing in the former part of this letter, I fhall content myfelf with adducing fome additional information, and fuch as, I hope, will convince Mr. C. that my ideas are not hypothetical.

The daughter of —— Burnham, a labouring man, at Quaenton, near this town, ætat. 6, was attacked on the 14th of November with the ufual fymptoms of typhus. As I did not fee the child till the 17th, I cannot pofitively declare, whether fhe had any thing previous to my vifiting her, but at this time her pulfe was languid and unequal, tongue black and dry, and a confiderable number of petechiæ made their appearance on the breaft. She was then ordered whatever quantity of red wine could be obtained from parochial affiftance, (which I need not obferve, is in general fcanty,) and had a pint of ftrong decoction of bark, with tincture and extract, and aromatics, acidulated with the acidum vitriolicum, and bark powders to be taken in the intermediate times. I heard the next morning, fhe was much the fame as when I faw her, and defired they would perfevere in the ufe of the bark and wine. I vifited her on the 19th, and found her in a delirium, which had continued from the night preceding. The bark was entirely rejected, jected, and nothing would remain on the ſtomach but red wine, of which ſhe had taken about three quarters of a pint. The petechiæ were not increaſed, but aſſumed a more purple appearance, and the fæces were regularly diſcharged, but quite black and offenſive. As the dernier reſort, I recommended them to procure ſome ſmall-beer yeaſt, and having made an infuſion of malt, to give a large ſpoonful of the former, diluted with the latter, every four hours. This method was regularly perſiſted in, and I had the pleaſure to hear the next afternoon, ſhe had paſſed the preceding night much better, and the delirium was ſomewhat abated. 21ſt. I was much pleaſed to obſerve the appearance of my little patient; ſhe was ſenſible, and gave me her hand on approaching the bed-ſide. The yeaſt was continued as before, and the pariſh officers thinking her out of danger, refuſed to continue the wine; but, on application to the worthy Rector of the village, he generouſly provided her with a bottle. The next day her father informed me ſhe was ſo much better, I need not go to Quaenton; and as I heard no more from him, I did not call till the 26th, when ſhe was ſo far recovered, as to be free from any complaint except debility.

It is worthy obſervation, that the ſon of this man was afflicted with the ſame diſeaſe a month before, and had large and repeated quantities of bark and wine, with acids, but he died in a fortnight. This I mention, as it occurred at nearly the ſame time, and in the ſame family; the particulars of which are well known to the worthy Rector. It will be needleſs to particularize every ſymptom; I ſhall therefore briefly ſtate the moſt remarkable incidents.

E. Gibbons, a poor woman of this town, was ill during a week with putrid fever, and the treatment with bark, &c. ſeemed entirely uſeleſs; but on uſing the yeaſt, and infuſion of malt, *ſhe was out of danger in four days!*

The daughter of Mr. M——ly, of this town, ætat. 5, was exceedingly ill for twelve days, and was treated with bark, red wine, and brandy, but in a ſtate of approaching diſſolution; *one tea-cup full of yeaſt only appeared to reſtore her!*

Mr. S———y, of Claydon, and his child, ætat. three years, were now given over by an eminent phyſician, but were providentially ſaved by the timely adminiſtration of yeaſt.

From theſe ſtatements will it not appear ſatisfactory to a candid mind, that the early adoption of yeaſt not only accelerates the criſis, but is productive of a ſucceſsful termination. It has had to combat with the very worſt caſes, and is moſt generally poſtponed till after the exhibition of bark; and in

 numberleſs numberlefs inftances, where death appeared inevitable, it has reftored the fufferer with unexampled rapidity. I would recommend Mr. C. to perufe an ingenious pamphlet lately publifhed, and entitled, " A fhort Account of the infectious Malignant Fever, as it appeared at Uxbridge and its vicinity in the year 1799, and where the antiputrefcent influence of yeaft was peculiarly confpicuous. The intelligent author informs the public, " *he has found it particularly ufeful in a great number of cafes, and therefore cannot avoid recommending it as a moft powerful antiseptic in malignant fevers.*" In the cafe recorded by Mr. Brown, in No. XVI. the return of health, after fuch fevere indifpofition, was truly remarkable; and Dr. Lewin, in his ingenious letter, obferves, " IN A FEW DAYS, on the comparifon, I had the pleafure to obferve fome of our little patients much benefited by it, and was particularly ftruck with the fpeedy convalefcence of one whofe fymptoms had been moft diftreffingly ftationary." Alfo, in an Epiftle I had the pleafure to receive from the Doctor, he adds, (what will be found explanatory of the former cafe publifhed by me in No. XXI. which, on account of the fpeedy relief experienced, is objected to by Mr. C. as vague and unfatisfactory) " I feel confident in afferting, that I have witneffed more examples of true typhus arifing at their acme in feven or eight days fince the adoption of this remedy than formerly;" and concludes, with affuring me, that " it has been employed by the moft judicious in that neighbourhood, and is extenfively ufed in a part of Staffordfhire, Worcefter, and Shropfhire."

Mr. C. has deemed my former letter unworthy a man, who profeffes to be fearching after truth; but how my actions have juftified fuch a conclufion, I am at a lofs to conjecture. It has been from a wifh to ferve that invaluable caufe that I have ventured to obtrude on the public attention, knowing that oppofition is the fureft teft of truth; and facts cannot be more firmly eftablifhed, than when affailed by refellible enquiry. I have made no claim to difcovery; nor have my views been prompted by ought but humanity and a love of inveftigation; and I am inclined to believe, if Mr. C. rightly confiders, he will lofe a portion of that refentment he apparently feels. It is evident I have not built my expectations on fpeculative notions, or been amufed with the illufion of hypothetical doctrine; for in all cafes where a putrid tendency has manifefted itfelf, I have found the happieft effects from its ufe, particularly as a poultice in foul putrid ulcers.

One objection made to my letter, is obviated by the explatory note of Dr. Bradley, as it appears that wine cannot be fuppofed fraught with more effential fervice than fupporting the vis vis vitæ. As Mr. C. has conducted his remarks on the whole in a fair manner, and avoided thofe perfonal reflections too frequent in controverfial difcourfes, I am happy to elucidate his enquiries, and willing at all times to anfwer any reafonable doubts, when conducted in a liberal manner. Shielded by the irrefiftible banner of truth, I can bid defiance to the fhafts of malevolence, and oppofe with fafety the language of experience to miffile weapons, that will only recoil on their authors. Happily for mankind thefe important facts have been long known, and will eventually be in general efteem; but, like all introductions into the fcience of medicine, it will have an hoft of enemies to contend with. So much does mercenary inclination on the one hand, and bigotry on the other, retard the progrefs of information.

Having no more defire than Mr. C. to intrude on your attention, I fhall take my leave of this fubject, concluding in the words of Montaigne: "I have here a nofegay of culled flowers, and have brought nothing of my own but the thread that ties them."

I have the honour to be, &c.

*Winflow, Feb.* 9, 1801. J. H. GROSE.

---

