# Errata

**Published:** 1801-04

**Authors:** 


					"a?e 275> line 38, inftead of 'were now giver, over, read were Riven
over, 6

				

